# Method of the Determination of Exterior Orientation of Sensors in Hilbert Type Space †

**DOI:** 10.3390/s18030891

**Published:** 2018-03-17

**Authors:** Grzegorz Stępień

**Affiliations:** Institute of Geoinformatics, Żołnierska 46, Maritime University of Szczecin, 71-250 Szczecin, Poland; g.stepien@am.szczecin.pl

**Keywords:** isometric transformation, big rotation angles, similarity transformation, Total Free Station—TFS, oblique orthogonal coordinate system, exterior orientation of sensor, Multi-Centroid Isometric Transformation—MCIT, geodesy surveying, close range photogrammetry, Multi-Centroid Transformation—MCT

## Abstract

The following article presents a new isometric transformation algorithm based on the transformation in the newly normed Hilbert type space. The presented method is based on so-called virtual translations, already known in advance, of two relative oblique orthogonal coordinate systems—interior and exterior orientation of sensors—to a common, known in both systems, point. Each of the systems is translated along its axis (the systems have common origins) and at the same time the angular relative orientation of both coordinate systems is constant. The translation of both coordinate systems is defined by the spatial norm determining the length of vectors in the new Hilbert type space. As such, the displacement of two relative oblique orthogonal systems is reduced to zero. This makes it possible to directly calculate the rotation matrix of the sensor. The next and final step is the return translation of the system along an already known track. The method can be used for big rotation angles. The method was verified in laboratory conditions for the test data set and measurement data (field data). The accuracy of the results in the laboratory test is on the level of 10^−6^ of the input data. This confirmed the correctness of the assumed calculation method. The method is a further development of the author’s 2017 Total Free Station (TFS) transformation to several centroids in Hilbert type space. This is the reason why the method is called Multi-Centroid Isometric Transformation—MCIT. MCIT is very fast and enables, by reducing to zero the translation of two relative oblique orthogonal coordinate systems, direct calculation of the exterior orientation of the sensors.

## 1. Introduction

To describe the position of material points in three-dimensional Euclidean space, orthogonal coordinate systems are usually used. The selection of the system is free and dictated by practical reasons [1]. The transformation between two orthogonal coordinates system is also required quite commonly. In the fields of geodesy and photogrammetry the transformation through similarity is commonly used as well. This allows the transformation of coordinates between two relative oblique orthogonal systems. This takes place in engineering geodesy in which the measures are transformed to the coordinate system of the measured object or for example tunnel boring machines [2]. The transformation through similarity covers the scale change (homothetic) and isometry, i.e., translation and rotation [3]. The transformation through similarity often assumes that the set of points (space) behaves in accordance to the rigid body mechanics [4]. In this way the geophysical researches on tectonic plate movements are described [5]. 

If the molecular system (rigid body), which is originated in the zero intercept, is put into a rotary motion, after some time (t) the position on the OXY plane of its particular points in relation to the reference system, which is constant, can be described as the following relation (1) [6]:

(1) $$X=X^{\prime }\cos\omega t-Y^{\prime }\sin\omega t\;Y=X^{\prime }\sin\omega t+Y^{\prime }\cos\omega t$$

where:

- ω—angular velocity,
- t—time,
- X, Y—coordinates in the primary coordinate system (fixed),
- X’, Y’—coordinates in the secondary coordinate system.

**Figure 1:**
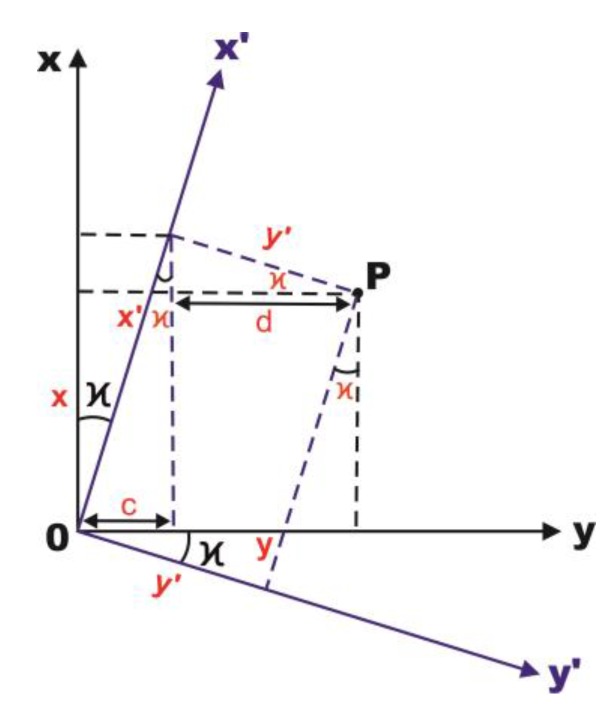
The transformation through rotation—the rotation in the oxy plane at *κ* angle—rotation around the z axis (yaw angle).

If a rectilinear motion is added to a rigid body moving with a uniform rotary motion, at the same time assuming the cycle time and constant velocity, what was described as relation (2), the relation (1) will be described as the following matrix (3) (Figure 1).

(2) $$\dot{\omega }=0,\;\dot{X}=0,\;\dot{Y}=0,\;\Delta t=1\;\left[s\right],\;\omega \cdot t=\kappa$$

where:

- A˙=∇t=∂∂t—magnitude gradient with respect to time,
- κ—angle of the coordinate system rotation in the OXY plane.
  (3) $$\left[\begin{array}{c} X\\ Y \end{array}\right]=\left[\begin{array}{c} X_{0}\\ Y_{0} \end{array}\right]+\lambda \cdot \begin{array}{cc} \cos\kappa & -\sin\kappa \\ \sin\kappa & \cos\kappa \end{array}\left[\begin{array}{c} X^{\prime }\\ Y^{\prime } \end{array}\right]$$

where:

- X_0_, Y_0_—the translation vector,
- λ = 1—scale change coefficient equal to one.
- other signs are assumed as in the relations (1), (2).

Indeed the transformation on the plane described by Equation (3) is the Helmert active transformation, shown in Figure 1, but in passive form, a transformation known in the fields of geodesy and photogrammetry [7]. However, due to the small difference it should be called a special example of the Helmert Transformation as the change scale coefficient λ is equal to 1. Thus, the transformation through similarity gets reduced to the isometric transformation consisting of translation and rotation, what is also common for surveying [8].

In the transformations through similarity which are connected to move, three rotation angles are used for the description, for example Taita-Bryan or Euler angles [9]. In geodesy and photogrammetry, the 3D transformation is described with relation [10]:

(4) $$\left[\begin{array}{c} X\\ Y\\ Z \end{array}\right]=\left[\begin{array}{c} X_{0}\\ Y_{0}\\ Z_{0} \end{array}\right]+\lambda \cdot A\;\left[\begin{array}{c} X^{\prime }\\ Y^{\prime }\\ Z^{\prime } \end{array}\right]$$

where:

- X, Y, Z—coordinates in the primary reference system,
- X’, Y’, Z’—coordinates in the secondary reference system,
- X_0_, Y_0_, Z_0_—the translation vector (movement of the primary coordinate system),
- λ—the factor of the scale change.
  (5) $$A=\left[\begin{array}{ccc} a_{11} & a_{12} & a_{13}\\ a_{21} & a_{22} & a_{23}\\ a_{31} & a_{32} & a_{33} \end{array}\right]=\left[\begin{array}{ccc} 1 & 0 & 0\\ 0 & \cos\omega & \sin\omega \\ 0 & -\sin\omega & \cos\omega \end{array}\right]\;\left[\begin{array}{ccc} \cos\varphi & 0 & -\sin\varphi \\ 0 & 1 & 0\\ \sin\varphi & 0 & \cos\varphi \end{array}\right]\left[\begin{array}{ccc} \cos\kappa & \sin\kappa & 0\\ -\sin\kappa & \cos\kappa & 0\\ 0 & 0 & 1 \end{array}\right]$$

where:

- a_11_, a_12_, a_13_, ..., a_33_—factors of the A rotation matrix,
- ω—the rotation around the X axis (clockwise), the roll angle,
- φ—the rotation around the Y axis (clockwise), the pitch angle,
- κ—the rotation around the Z axis (clockwise), the yaw angle.

The transformations are described by Equations (4) and (5) but in plenty of cases and for calculation convenience they are often reduced to infinitesimal transformations. Taking into account Equation (6) allows one to obtain the A matrix in the form (7):

(6) sinδ=δ, cosδ=1

where:

- δ—infinitesimal dimension (tending to zero) expressed in radians:
  (7) $$a=\left[\begin{array}{ccc} 1 & \kappa & -\varphi \\ \omega \varphi -\kappa & \omega \varphi \kappa +1 & \omega \\ \varphi +\omega \varphi & -\varphi \kappa -\omega & 1 \end{array}\right]≅\left[\begin{array}{ccc} 1 & \kappa & -\varphi \\ -\kappa & 1 & \omega \\ \varphi & -\omega & 1 \end{array}\right]$$
  where:
- ω, φ, κ—angles described as in (5) but expressed in radians.

By that means the matrix A described with the relation (5) is often brought to so called small rotation angles [11] (7). Consequently, this leads to the infinitesimal transformation [6] of the relation (4) to:

(8) $$\left[\begin{array}{c} X\\ Y\\ Z \end{array}\right]=\left[\begin{array}{c} X_{0}\\ Y_{0}\\ Z_{0} \end{array}\right]+\lambda \left[\begin{array}{ccc} 1 & \kappa & -\varphi \\ -\kappa & 1 & \omega \\ \varphi & -\omega & 1 \end{array}\right]\;\left[\begin{array}{c} X^{\prime }\\ Y^{\prime }\\ Z^{\prime } \end{array}\right]$$

The transformation described with the relation (8) often applies to aerial surveying. The situation changes when it comes to close range photogrammetry. The rotation angles are much larger than in the traditional aerial surveying so special algorithms must be used [12].

The transformations using matrix (7) are commonly used to determine the orientation of the reference ellipsoid and 3D base line [2]. For these purposes the Bursa-Wolf [13,14] transformations, described by the relation (9), and Molodensky-Badekas transformations [2], described by relation (10), are used:

(9) $$\left[\begin{array}{c} X\\ Y\\ Z \end{array}\right]=\left(1+\mathrm{ds}\right)\left[-\begin{array}{ccc} 1 & \epsilon_{Z} & -\epsilon_{Y}\\ \epsilon_{Z} & 1 & \epsilon_{X}\\ \epsilon_{Y} & -\epsilon_{X} & 1 \end{array}\right]\;\left[\begin{array}{c} X^{\prime }\\ Y^{\prime }\\ Z^{\prime } \end{array}\right]+\left[\begin{array}{c} t_{X}\\ t_{Y}\\ t_{Z} \end{array}\right]$$

where:

- $\epsilon_{Z}$—angle expressed in radians and marked the same as κ in the relation (5),
- $\epsilon_{Y}$—angle expressed in radians and marked the same as φ in the relation (5),
- $\epsilon_{X}$—angle expressed in radians and marked the same as ω in the relation (5),
- 1+ds—change scale coefficient,
- $t_{X},\;t_{Y},\;t_{Z}—$coordinates of the translation vector,
- other signs as in the relation (4).
  (10) $$\left[\begin{array}{c} X\\ Y\\ Z \end{array}\right]=\left(1+\mathrm{ds}\right)\left[-\begin{array}{ccc} 1 & \epsilon_{Z} & -\epsilon_{Y}\\ \epsilon_{Z} & 1 & \epsilon_{X}\\ \epsilon_{Y} & -\epsilon_{X} & 1 \end{array}\right]\;\left[\begin{array}{c} \bar{X}\\ \bar{Y}\\ \bar{Z} \end{array}\right]+\left[\begin{array}{c} t_{X}\\ t_{Y}\\ t_{Z} \end{array}\right]$$
  where:
- $t_{X},\;t_{Y},\;t_{Z}—$coordinates of the translation vector measured accordingly along the axis $X^{\prime },\;Y^{\prime },\;Z^{\prime }$(secondary coordinate system),
- $\bar{X},\;\bar{Y},\;\bar{Z}$—coordinates of the translation vector of the secondary coordinate system to the common centroid of the reference systems,
- other signs as in the relation (9).

The transformations described by the relations (9) and (10) are based on the rotation matrix (7) which is determined for small rotation angles. Moreover, in the Moldensky-Badekas transformation the displacement of both systems to the common centroid occurs. 

Moreover, in similarity (and isometric) transformations described by relations (5), (8), (9), (10) angular elements of exterior orientation of sensor, expressed in the rotation matrix, are involved with displacement of the coordinate system and it cannot be calculated separately. Considering the above mentioned relations the following main question (research problem) arises: is it possible to build such a space in which:

1. translations between two relative oblique orthogonal coordinate system are reduced to zero;
2. simultaneously the angular relative orientation of both coordinate systems is constant;
3. it is possible to direct computing the rotation matrix separately from displacement of the system and from the scaling factor.

Answering this question—OUR main research problem—permits on direct determination of the rotation matrix and then exterior orientation of a sensor without connection to displacement of the coordinate system. Solving such a problem is the purpose of this publication. 

The remainder of this paper propose an algorithm based on the use of three (or more) centroids and the generation of the new space of points in which the following elements will be calculated: change scale coefficient (checking the similarity transformation), exterior orientation of sensor: rotation matrix and coordinates of sensor expressed in external coordinates system. The proposed method, unlike the Moldensky-Badekas transformation or Bursa-Wolf transformation, can be applied for big rotation angles, and for close range photogrammetry purposes. The method is a further development of the Total Free Station (TFS) transformation [15] to several centroids in Hilbert type space. It can be applied for local, precise surveying as well as for geodynamic, close range photogrammetry and engineering purposes, especially for non-stable places (e.g., on floating vessels) where levelling of an instrument may not be possible and it is necessary to determine the exterior orientation of sensors. This method is suitable for sensors which can measure angles and/or distances like Total Station, laser scanners (e.g., TLS—Terrestrial Laser Scanner, ALS—Airborne Laser Scanner), echo-sounders and also can be applied to metric and non-metric cameras for photogrammetric purposes.

## 2. Materials and Methods 

The following article presents the algorithm of the transformation through similarity (isometric) based on the transformation in the newly normed R^3^ Euclidean space. An Euclidean space is a R^n^ vector space with Euclidean scalar product and norm and metric [16]. The linear space, based on which the norm is defined (one or more), is called the normed space. In linear normed space the length of the vector is determined by the real-valued functional called also a norm of the space that meets the axioms of norm [17]:

(1) ‖x‖≥0 where ‖x‖=0⟺x=0 (positive definiteness)
(2) ‖x+y‖≤‖x‖+‖y‖ (fulfilling the triangle condition)
(3) ‖αx‖=|α|·‖x‖ (first degree homogeneity)

Every scalar product in the vector space creates a norm of vector (11). Each norm in the vector space determines a metric as a norm of the difference of u and v vector (12) [16]:

(11) $$\|u\|=\sqrt{\left(u,u\right)}$$

(12) d(u,v)=‖u‖−‖v‖

Thus, the norm must be perceived as the general length of the u vector (11) and the metric in the normed space must be perceived as the d distance between a pair of the elements of the space (distance between the pair of points) (12). The following considerations are carried out in the complete linear vector space (Banach space), normalized space, in which each Cauchy sequence of its elements converges to an element of this space [18]. Thanks to the possibility to determine the scalar product that generates the norm of vectors, the space is an unitary space (i.e., a vector space with scalar product), defined as the Hilbert space. The R^3^ normed Euclidean space with standard—Euclidean scalar product is a subset of Hilbert space in which the considerations are carried out. Accordingly, in the present discussion, the key element of the definition of the newly normalized Hilbert space is the definition of its norm, i.e., the method of determining the length of vectors in the space.

**Figure 2:**
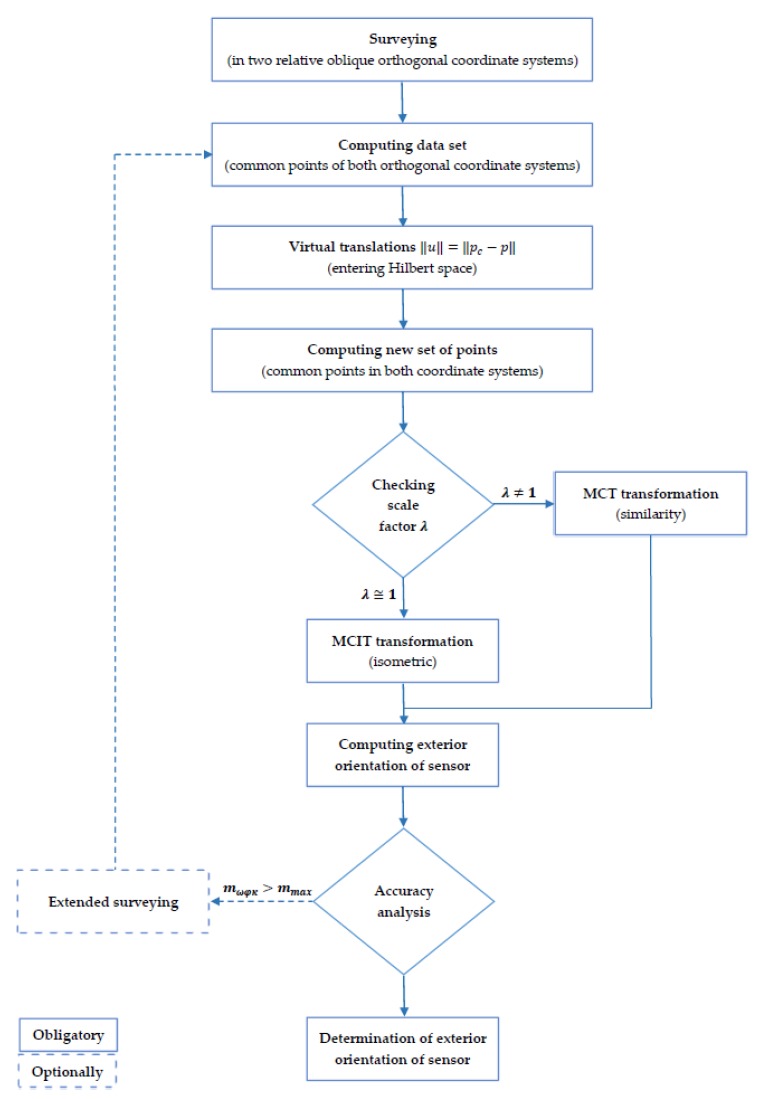
Algorithm of determination of exterior orientation of sensors.

This publication proposes the algorithm for the transformation through similarity (isometric) given by Equation (13) and presented in Figure 2. Problem is solved in a R^3^ normed Euclidean space which is defined as the Hilbert type space through reduced to zero translations between two relative oblique orthogonal coordinate system by the norm space.

In this publication the transformation through similarity is given by the relation:

(13) $$\left[\begin{array}{c} X^{\prime }\\ Y^{\prime }\\ Z^{\prime } \end{array}\right]=\left(\lambda \cdot \;\left[\begin{array}{c} X\\ Y\\ Z \end{array}\right]+\left[\begin{array}{c} {\bar{X}}_{0}\\ {\bar{Y}}_{0}\\ {\bar{Z}}_{0} \end{array}\right]\right)\cdot A$$

where:

- ${\bar{X}}_{0},\;{\bar{Y}}_{0},\;{\bar{Z}}_{0}$—the translation vector (movement of the primary coordinate system),
- other signs are assumed as in the relation (4).

In the transformation described by relation (13), which in the current paper is called the Multi-Centroid Transformation (MCT), scaling the primary coordinate system comes first. In case the scaling factor λ≅1, then the transformation becomes the isometric transformation and is called Multi-Centroid Isometric Transformation (MCT). Both MCT and MCIT are development of the TFS transformation [15] to the case of at least three centroids. The next step is its translation and the last element is the rotation expressed in the A rotation matrix.

The proposed algorithm for such a problem of transformation, with conditions:

1. translations between two relative oblique orthogonal coordinate system are reduced to zero;
2. simultaneously the angular relative orientation of both coordinate systems is constant;
3. it is possible to directly compute the rotation matrix and simultaneously separately from the displacement of the system and the scaling factor;

is solved in Hilbert space, according to algorithm presented on Figure 2. This transformation is the inverse transformation of (13) and is given by the relation:

(14) $$\left[\begin{array}{c} X\\ Y\\ Z \end{array}\right]=\left(\frac{1}{\lambda }\cdot \;\left[\begin{array}{c} X^{\prime }\\ Y^{\prime }\\ Z^{\prime } \end{array}\right]\cdot A^{-1}\right)-\left[\begin{array}{c} {\bar{X}}_{0}\\ {\bar{Y}}_{0}\\ {\bar{Z}}_{0} \end{array}\right]$$

where the signs are as in relation (13).

**Figure 3:**
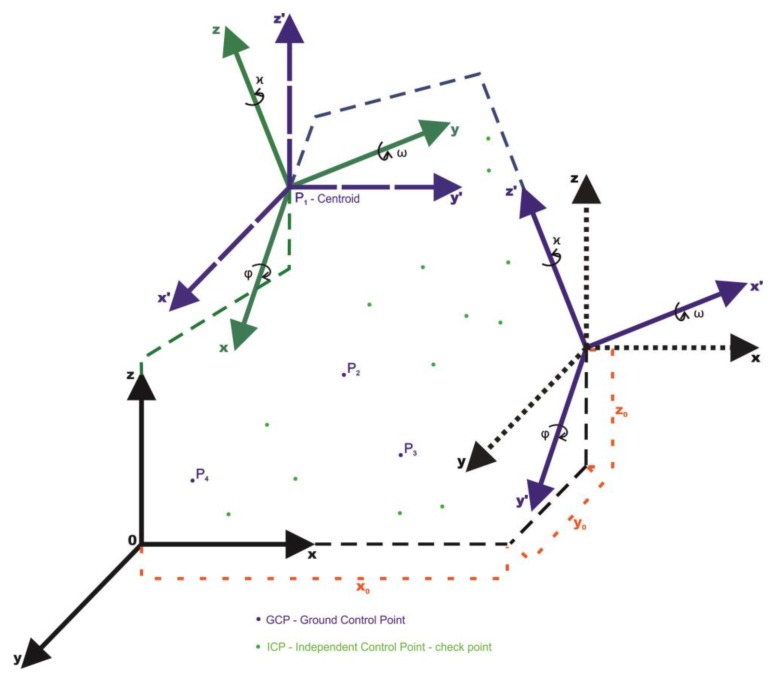
The transformation with the use of a centroid according to the relation (13)—development of TFS transformation [15].

**Figure 4:**
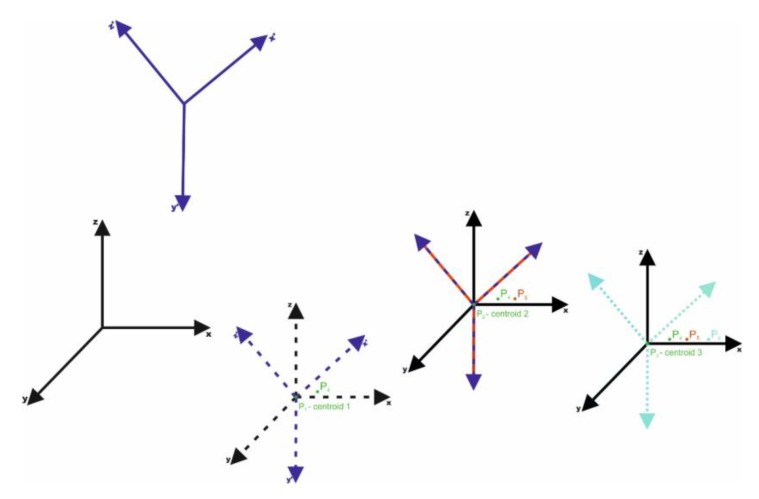
Multi-Centroid Transformation (MCT) with use of three centroids.

The proposed solution for such a problem is to start, after surveying and collecting a data set, with the displacement of the coordinate systems—primary and secondary systems—to one common centroid (Figure 3). Each coordinate system is moved along its axis so the relative angle orientation of both systems does not change. This action is repeated for (at least) three times for three (or more) centroids (Figure 4). In this way each of the displacements generates new points (Table 1). 

The points are known in both coordinate systems and have a function as centroids (points on which the systems are displaced. The situation of displacing the points on four centroids—four points already known in both relative oblique and scale (by any factor) coordinate systems (Figure 3)—can be considered. By displacing the systems on P1 point—the first centroid—three new pairs of points (vectors) are obtained in both the oblique and scale coordinate systems: P2 and P2′, P3 and P3′, P4 and P4′. By displacing on the second centroid, three pairs of points will be obtained once again. P1 and P1′, P5 and P5′, P6 and P6′, where $P_{1}=-P_{2}$ and ${P^{\prime }}_{1}=-{P^{\prime }}_{2}$. As there are only two pairs of points—two new vectors are linearly independent in relation to the ones generated from the P1 centroid. By displacing the systems on the third centroid, three pairs of points will be obtained: P7 and P7′, P8 and P8′, P9 and P9′, where only the pairs of P9 and P9′ are linearly independent. The displacement to the fourth centroid generates vectors opposite to the displacement on the first one After all the displacements the following pairs of points (vectors) are obtained and linearly independent: P2 and P2′, P3 and P3′, P4 and P4′, P5 and P5′, P6 and P6 as well as P7 and P7′. The data are presented in the Table 1 and the linearly independent vectors are marked with bold font. 

For four centroids and with further displacements we obtain six new pairs of points in both the relative oblique and scaled coordinate systems. The fourth displacement does not generate a new pair of points. The number of generated pair of points (vectors) in the new space can be defined as follows:

(15) $$S_{n}=\sum_{k=1}^{k=n-1}\left(n-k\right)$$

where S_n_—number of generated pair of points (vectors), and n—number of centroids.

According to the relation (15) it can be calculated that every time n−1 of points are generated, where n means the number of centroids. This is because the last centroid generates no new points, all of them are opposite to the points generated on previous centroids. For three centroids (n = 3), three new pairs of points are obtained. Thus, to calculate the rotation matrix (for big rotation angles) and the change scale coefficient, four centroids are necessary that will allow to obtain six new pairs of points in Hilbert space. 

**Figure 5:**
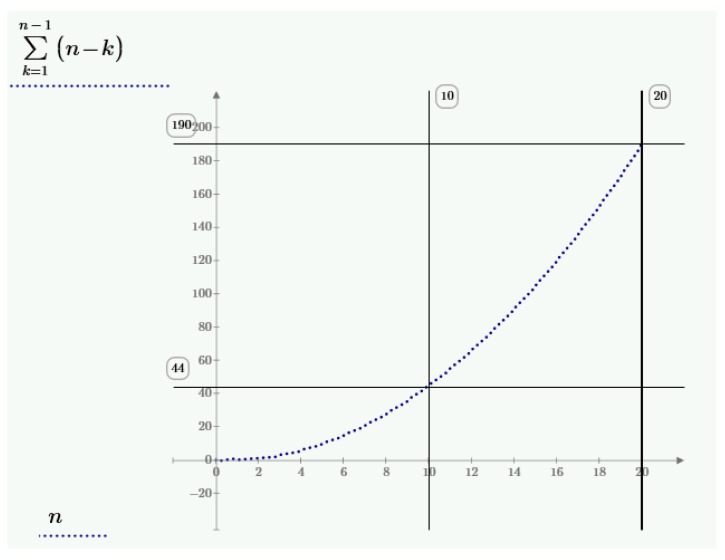
The number of points in the centroid function in accordance to the relation (14).

For example, 44 new pairs of points can be generated with only 10 centroids and 190 new pairs of points can be generated with 20 centroids. The relation (15) of the new pair of points to the number of centroids is presented in Figure 5.

The norm of Hilbert space, where the points for MCT are generated, due to the fact that the space is linear, is defined by the length of the vector (real-valued functional) which is described by relation (11). The scalar product of the vector (cos angle = 1) defines the length of the vector. Thanks to the possibility to determine the vector product in the linear space, in which the considerations are realized, the space is the inner product space (Hilbert space). The length of the u vector in the space is described by the relation (11). The u vector is determined by the relation:

(16) $$p+u=p_{c}$$

where:

- u—the vector in the normed Hilbert (R^3^) space, where its length is calculated in accordance to the relation (17) is the space’s norm;
- p—the vector originated in the zero intercept of the primary coordinate system and ends in the p point,
- $p_{c}$—the vector originated in the zero intercept of the primary coordinate system and ends in the p_c_ point which is the centroid.

Once the relation (16) is transformed we obtain the norm of the Hilbert (R^3^) space:

(17) $$\left\|u\|=\|p_{c}-p\right\|$$

The norm in the newly defined vector space produces a metric as the norm of the difference between the p_c_ and p vectors and is the “input” to this new space. The p point is any point and the p_c_ point is the centroid.

The points in the new Hilbert space are induced by the displacement of the systems. The displacement of the systems takes place (at least) three times, for four known points in both systems from which three become centroids (the fourth displacement, as mentioned before, does not generate new points). The translation of the systems takes place along the axis of each of the systems. The procedure does not change the relative angle orientation of the coordinate systems and at the same time allows one to generate points (vectors) in the new Hilbert space. In this way three pairs of symmetric vectors are received and thanks to this it is possible to calculate (check) the scale factor and rotation matrix. When determining the scale in the orthogonal transformation it is necessary to use the invariant of the square of the length of the vector in relation to the rotation, which is the invariant of the transformation [1]. In the transformation through similarity the system is scaled and especially if the change scale coefficient equals one the transformation becomes the isometric one. As a consequence, after displacing the systems (entering the new space—Hilbert space), the problem of changing the scale of the system and determining the rotation matrix is solved. In the next step the change scale coefficient is calculated using the fact that the scale does not change in relation to the transformation. Thus, the scale change does not result from the rotations of the system but from scaling the primary coordinate system. Thus, comparing the lengths of the vectors in the newly normed space coordinate system without the rotation and that in the system with the rotation, the ratio of their lengths will allow to determine the change scale coefficient λ from the dependence:

(18) $$\lambda =\frac{\left\|p^{\prime }\right\|}{\left\|p\right\|}$$

where:

- p—the vector in the newly normed space, originated in the zero intercept (without the rotation and scaling) and ended in the p point,
- $p^{\prime }$—the vector in the newly normed space, originated in the zero intercept (with the rotation and scaling) and ended in the p point.

The change scale coefficient can be determined from the relation (18) for many times and for several point and then averaged. It can be also determined once for the vector of the highest length in the rotated system.

Once the change scale coefficient is determined (checked that is equal one) it is possible to determine the rotation matrix from the relation (13). For this purpose the approach presented in [12] is used in the algorithm for big rotation angles where the condition on the orthogonality of the A matrix is used, what can be described as the relations (19), (20):

(19) $$A\cdot A^{T}=A^{T}\cdot A=I$$

where:

- A—the rotation matrix defined in analogy to the relation (5),
- I—the identity matrix.
  (20) $$a_{11}^{2}+a_{12}^{2}+a_{13}^{2}=1\;a_{21}^{2}+a_{22}^{2}+a_{23}^{2}=1\;a_{31}^{2}+a_{32}^{2}+a_{33}^{2}=1\;a_{11}^{2}\cdot a_{12}^{2}+a_{21}^{2}\cdot a_{22}^{2}+a_{31}^{2}\cdot a_{32}^{2}=0\;a_{11}^{2}\cdot a_{13}^{2}+a_{21}^{2}\cdot a_{23}^{2}+a_{31}^{2}\cdot a_{33}^{2}=0\;a_{12}^{2}\cdot a_{13}^{2}+a_{22}^{2}\cdot a_{23}^{2}+a_{32}^{2}\cdot a_{33}^{2}=0$$

Once the A matrix is determined, the rotation of the system takes place. This leads to displacement of the axes of the both systems—the primary and secondary coordinate systems. The next and last step in the MCT (MCIT) is translation (returning one) of the systems along the already known track as it is the same track the system without the rotation was translated to the first centroid in accordance to the relation (21). Therefore, this is a translation of a known vector with coordinates opposite to the coordinates of the first centroid on which the system without the rotation was displaced.

(21) $$P=P^{\prime }-P_{c2}$$

where:

- P—the matrix of the points transformed from the secondary coordinate system to the primary coordinate system,
- $P^{\prime }$—the matrix of the point after scaling and rotating in the newly normed Hilbert space,
- $P_{c2}$—the translation matrix (returning one) to the real-valued space, where the measurements were carried out—the matrix consisting of vector opposite to the centroid no. 2.

In this way and in accordance to relation (21), the opposite translation and return to the real-valued space, where the measurements were carried out, take place. In the next step the errors on the check points (ICT—Independent Control Points) are calculated, which coordinates in both systems (the one with the rotation and the one without the rotation) were known but these were not the centroids and did not take part in the transformation. Then the mean errors of coordinates are calculated (standard deviations) once the transformation is finished. 

The discussion on the results may be also carried out base on the maximal errors of coordinates, which are three times bigger than mean errors (according to Gaussian distribution with confidence level of 99.73%). Then maximal errors of exterior orientation accuracy of a sensor which is connected to the coordinates accuracy can be estimated, for infinitesimal values of errors, according the same relation (13), as follows:

(22) $$\left[\begin{array}{c} M_{X^{\prime }}\\ M_{Y^{\prime }}\\ M_{Z^{\prime }} \end{array}\right]=\left(M_{\lambda }\cdot \left[\begin{array}{c} M_{X}\\ M_{Y}\\ M_{Z} \end{array}\right]+\left[\begin{array}{c} M_{X_{0}}\\ M_{Y_{0}}\\ M_{Z_{0}} \end{array}\right]\right)\cdot M_{A}$$

where:

- M—maximum error according to Gaussian distribution with confidence level of 99.73%,
- other signs as in the relation (13).

The relation (22) can be present in parametric form:

(23) $$M_{X_{i}^{\prime }}=\left(M_{\lambda }\cdot M_{X_{i}}+M_{X_{0}^{\prime }}\right)\cdot M_{A}$$

where:

- $M_{X_{i}^{\prime }}=\left[\begin{array}{c} M_{X^{\prime }}\\ M_{Y^{\prime }}\\ M_{Z^{\prime }} \end{array}\right],$ $M_{X_{i}}=\left[\begin{array}{c} M_{X}\\ M_{Y}\\ M_{Z} \end{array}\right]$, $M_{X_{0}^{\prime }}=\left[\begin{array}{c} M_{X_{0}}\\ M_{Y_{0}}\\ M_{Z_{0}} \end{array}\right]$,
- other signs as in the relations (22) and (13).

Relation (23) can be modified to:

(24) $$M_{A}={\left(M_{\lambda }\cdot M_{X_{i}}+M_{X_{0}^{\prime }}\right)}^{-1}\cdot M_{X_{i}^{\prime }}$$

Separate values for the angular exterior orientation of sensor can be calculate on the basis of relation (5), for errors—relation (24), as follows:

(25) $$\varphi =\mathrm{arc}\sin\left(-a_{13}\right),\;\omega =\mathrm{arc}\tan\left(\frac{-a_{23}}{a_{33}}\right),\;\omega =\mathrm{arc}\tan\left(\frac{-a_{23}}{a_{33}}\right)$$

where:

- φ, ω, κ—external angular orientation of sensor expressed in radians,
- signs a_13_, a_23_, a_33_—factors of the A rotation matrix—as in the relations (5).

Simultaneously for infinitesimal values of errors in relation (24) $M_{X_{i}}$ is equal zero, and $M_{\lambda }$ is equal one, it is received the isometric transformation with simplification $M_{X_{0}^{\prime }}\approx M_{X_{i}^{\prime }}$ than $M_{A}$ matrix can be simplify to relation:

(26) $$M_{A}={\left(M_{X_{i}^{\prime }}\right)}^{-1}\cdot M_{X_{i}^{\prime }}$$

Relation (26) can be applied for infinitesimal values of errors e.g., in case of geodetic surveying carried out with the levelled instrument. The rotation errors around the X axis (∂ω) and Y axis (∂φ) are mostly related to the instrument levelling errors, while the rotation error around the Z axis (∂κ) is related to the accuracy of the instrument (if the geometrical and axial conditions are met). 

Based on the known errors of the 3D base line coordinates—in relation (24) matrix $M_{X_{i}}$—the estimated errors of the exterior orientation of sensors can be calculated at first. From this point of view knowing points and its errors in primary coordinate system and precision of sensor which will be used in oblique orthogonal coordinate system makes possible to estimate expected values of errors of exterior orientation of sensor or calibrate instrument IMU (Inertial Measurements Unit) through the check correctness of results against values calculated as differentials of (24) through relations:

(27) $$\partial \varphi =\frac{1}{\sqrt{1-a_{13}{}^{2}}}\cdot \partial a_{13},\;\partial \omega =\frac{1}{1+{\left(\frac{a_{23}}{a_{33}}\right)}^{2}}\cdot \partial \left(\frac{a_{23}}{a_{33}}\right),\;\partial \kappa =\frac{1}{1+{\left(\frac{a_{12}}{a_{11}}\right)}^{2}}\cdot \partial \left(\frac{a_{12}}{a_{11}}\right)$$

where:

- ∂—sign of differential,
- other signs as in the relations (24) and (5).

Taking into account, on the relation (5) basis, that:

(28) $$-a_{13}=\sin\varphi ,\;\frac{-a_{23}}{a_{33}}=\tan\omega ,\;\frac{-a_{12}}{a_{11}}=\tan\kappa$$

for infinitesimal values in relation (6), relation (28) can be expressed as the identity in the form:

(29) $$\partial \varphi \equiv \partial a_{13},\;\partial \omega \equiv \partial \left(\frac{a_{23}}{a_{33}}\right),\;\partial \kappa \equiv \partial \left(\frac{a_{12}}{a_{11}}\right)$$

## 3. Results

Within the experiment a few lab (numeric) and field researches were performed. In laboratory tests, the mathematical correctness of the proposed method on the assumed data that were used in the matrix was checked (all the following data were assumed in [m]). In Table 2 below are shown detailed calculations for one data set.

In the laboratory data set it was assumed that the coordinates are with a precision of ±1 mm. Next the system (set of points) was scaled. The assumed change scale coefficient equaled λ = 1257, and the points were translated of the vector [1555.555 154,000.321 −145.356] and rotated with the following angles: ω = 27.35478°, φ = 5.578938°, κ = 19.30716° (for calculation of A). In this way the matrix of points in the scaled, translated and rotated system was obtained as in Table 3.

Matrix A is calculated according to relation (5):

(30) $$A=\left[\begin{array}{ccc} 0.94192 & -0.32999 & -0.06242\\ 0.26659 & 0.84771 & -0.45860\\ 0.20425 & 0.41533 & 0.88645 \end{array}\right]$$

In the next step, the “entry” to the newly normed Hilbert (R^3^) space was performed according to the relation (17), by translating the systems to centroids (successively to the points—rows—no. 2, 7 and 1 in Table 4 and Table 5). Below in Table 4 and Table 5 the data after translation to the centroid no. 2 is presented (sequentially for primary and secondary systems):

Once the systems were displaced on the further centroids, the matrices of points in both systems (secondary system—rotated and scaled—P4, primary system—without the rotation and the scale change—p4) were generated:

(31) $$p4=\left[\begin{array}{cc} \begin{array}{cc} d_{19} & d_{17}\\ d_{29} & d_{27}\\ d_{39} & d_{37} \end{array} & \begin{array}{cc} e_{19} & f_{17}\\ e_{29} & f_{27}\\ e_{39} & f_{37} \end{array} \end{array}\right]=\left[\begin{array}{cc} \begin{array}{cc} -11.615 & -5.325\\ -15.581 & 25.502\\ -1.120 & 2.288 \end{array} & \begin{array}{cc} -6.290 & -8.064\\ -41.083 & 38.746\\ -3.408 & 2.288 \end{array} \end{array}\right]\;P4=\left[\begin{array}{cc} \begin{array}{cc} D_{19} & D_{17}\\ D_{29} & D_{27}\\ D_{39} & D_{37} \end{array} & \begin{array}{cc} E_{19} & F_{17}\\ E_{29} & F_{27}\\ E_{39} & F_{37} \end{array} \end{array}\right]=\left[\begin{array}{cc} \begin{array}{cc} -7201.278 & -17,062.375\\ -19,849.270 & 24,070.846\\ -12,364.317 & 14,495.949 \end{array} & \begin{array}{cc} 9861.097 & -25,798.865\\ -43,920.116 & 37,265.435\\ -26,860.266 & 20,706.943 \end{array} \end{array}\right]$$

Four homologous pair of points in matrixes p4 and P4 were generated from only two points no. 9 and no. 7. Points generated using individual centroids were signed as D (d)—for the first centroid, E (e)—for the second centroid, F (f)—for the third centroid. In the next step the scale change coefficient from the relation (18) was calculated for six newly generated points (vectors):

(32) $$\lambda_{\mathrm{av}}=\frac{\lambda_{27}+\lambda_{21}+\lambda_{72}+\lambda_{71}+\lambda_{12}+\lambda_{17}}{6}=1256.999999997$$

It is obvious from the above that the preciseness of determining the scale change coefficient is 10^−9^ of the input data, what indicates the correctness of the designation of the coefficient and also the confirmation of isometric transformation. In the nest step the rotation matrix was calculated on the basis of relation (13) with condition (20):

(33) $$N14=\left[\begin{array}{ccc} 0.9419191 & 0.26659 & 0.2042504\\ -0.3299875 & 0.8477106 & 0.4153252\\ -0.0624236 & -0.4586028 & 0.8864461 \end{array}\right]$$

by multiplying the N14 matrix with the A (rotation) matrix:

(34) $$A=\left[\begin{array}{ccc} 0.94192 & -0.32999 & -0.06242\\ 0.26659 & 0.84771 & -0.45860\\ 0.20425 & 0.41533 & 0.88645 \end{array}\right]$$

calculated for angled assumed in advance (ω = 27.35478°, φ = 5.578938°, κ = 19.30716°) of which the primary system was rotated and the unit matrix was obtained:

(35) $$N14\cdot A=\left[\begin{array}{ccc} 1.00000002 & -0.00000004 & 0.00000004\\ 0.00000002 & 1.00000002 & -0.00000002\\ 0.00000003 & -0.00000003 & 0.99999996 \end{array}\right]≅I$$

In the next step scaling and rotating of the opposite matrix (N14) were performed, in accordance to the relation (13), as well as the translation to the system from the newly normed Hilbert (R^3^) space to the real-valued space of a vector opposite to the centroid no. 2 (Equation (21)). The following deviations were obtained in the check points (ICP) (presented with overstated precision) in Table 6.

The mean errors (standard deviations) of the typical observation for example data sets are presented in Table 7.

The mean errors (standard deviations) of the typical observation for example data set distorted by random errors according to normal Gaussian distribution are presented in Table 14. Considering Table 7, Table 8 and Table 14 it was found that the method is mathematically correct and analogue calculations for field measures were carried out. 

The next step was field tests in a real surveying environment. All data for field measurements were given in [m]. The surveying by Total Station was performed in two relative oblique coordinate systems that were translated by an unknown value. Firstly, the measurements were carried out with the levelled and centered instrument with the compensator turned on. The following points were obtained (coordinates of the points) are presented in Table 9.

Then the instrument was not levelled and translated with the unknown vector. The coordinate system was connected with the translated and oblique instrument (Total Station). Then the compensator in the instrument was turned off. The field surveying was performed with the Total Station (model Trimble M3) with the angle accuracy of 2” and 2 mm + 2 ppm distance measurement accuracy measured to reflective foil. Nine points in two full series were measured. The distance between the points and the instrument was from 10 to 50 m. In this way a matrix of the points was obtained as in Table 10.

In the newly normed Hilbert space the scale change coefficient (18) was calculated for the lengths of six vectors in both coordinate systems:

(36) $$\lambda_{\mathrm{av}}=\frac{\lambda_{27}+\lambda_{21}+\lambda_{72}+\lambda_{71}+\lambda_{12}+\lambda_{17}}{6}=1.00004$$

Coordinates were measured with a Total Station (angles and distances from two different stations). Computed scale values reflect the random errors in the observation and also is connected to the accuracy of the Total Station itself. After checking the scale factor coefficient in the nest step the rotation matrix was calculated on the basis of relation (13) with condition (20). It was assumed the stochastic model of errors according to normal Gaussian distribution. The following average errors [mm] were obtained after transformation (Table 11 and Table 12).

In the next and last step, the exterior orientation of sensor and errors were calculated.

## 4. Discussion 

During the calculations of the laboratory data (numeric study) the MCT transformation was compared to the Moldensky-Badekas, Bursa-Wolf, Leica (algorithm applied in the Leica Total Station—PCMS) and Total Free Station transformations. Results of laboratory data processing are presented in Table 13 and Table 14. 

The analysis of Table 13 shows that the highest accuracy on laboratory data (perfect data)—without distortion by random errors, are obtained in MCT transformation. MCT is the development of the TFS transformation, TFS is the second in accuracy. The Leica transformation—PCMS—also presents high accuracy. The results are worth emphasizing considering fact that MCT was not calculated using the Least Squares Method and simultaneously is the most accurate method. All remaining methods were compensated by the Least Squares Method. 

Data presented in Table 14 were calculated on the basis that coordinates are distorted according to a normal Gaussian distribution. It was assumed that the standard deviation is equal for each coordinate, and the standard deviation calculated for the point was $\sigma_{z}=2.60\;\mathrm{mm}$. This value is close to calculated mean error after transformation $m_{P}=3.01\;\mathrm{mm}$. This shows that the MCT transformation can be applied also for data with random errors.

Data collected and transformed for field measurements in a real environment were also calculated using various transformation methods. In this case the accuracy of MCT is similar to that of the Moldensky-Badekas, Bursa-Wolf, and Leica (PCMS) transformations (Table 15). The worst accuracy is in TFS transformation, what can be caused by choosing only one centroid. In this case the MCT transformation was also calculated without using the Least Squares Method and all remaining methods were compensated by the Least Squares Method. In the MCT transformation the m_x_ and m_y_ values are smaller than in other methods but m_z_ is bigger. In such measurements like Total Station the Z value errors can be the biggest because of residual instrumental errors, such as collimation and inclination errors which were not improved in the MCT transformation using the Least Squares Method like in other methods.

Simultaneously obtained angular results (Table 8 and Table 12)—exterior orientation of sensor—are very accurate. The mean error in laboratory tests is in range of 2 × 10^−6^ degree and for field surveying is was about 0.5 × 10^−5^ degree. It proves that it is possible to obtain high measurement accuracies with a non-centered and oblique instrument (e.g., Total Station, Terrestrial Laser Scanner). Simultaneously it has to be emphasized that for high accuracy of the determination of exterior orientation of sensor it is necessary to precisely measure the 3D base line.

## 5. Conclusions

The current publication has proposed a new way of determining the exterior orientation of sensors. It is through transformation similarity and isometric Expression (13) and its solution with a Multi-Centroid (Isometric) Transformation (MCT, MCIT) in Hilbert type space. The presented method is based on the so called multiple virtual translation, with known in advance coordinate system translation, to the new vector space. The virtual translation produces points for calculating the rotation matrices and its accuracy. The operation is described by the space norm of the new space R^3^—Hilbert type space (17). In such a space the translation vector equals zero so it is possible to calculate the scale change coefficient directly by comparing the lengths of the vectors of the rotated and scaled system to the vectors of the system without the rotation. Then the rotation matrix, angular exterior orientation of sensor and its accuracy are calculated. Knowledge of four adjustment points is necessary for the transformation as they are useful for designating the virtual translations of centroids and generating the points to calculate the scale change coefficient and rotation matrices. The proposed method allows to enlarge the number of points necessary for the calculations by displacing on further centroids. Four points (centroids) generate six points for calculations. Ten centroids allow one to obtain 44 new points in the new space and twenty centroids allow one to obtain 190 points. It can improve the accuracy against traditional e.g., Helmert, Bursa-Wolf, Moldensky-Badekas transformations, especially for more than four adjusted points. Simultaneously the MCT transformation gives proper values for big rotation angles thus it can be applied for close range photogrammetry. In the MCT (MCIT) transformation there is no need to calculate a translations vector—it is equal to zero, as the new origin of the coordinate system is already known as a centroid.

During the calculations on the laboratory data (numeric study) the obtained accuracy of coordinates (standard deviations) equaled 10^−4^ of the input data for data without random errors and 10^−3^ for data distorted by random errors (on the same level of accuracy). This proved the mathematical correctness of the proposed method. Field studies for various data sets gave an accuracy that equaled 2.4–4.5 mm, what in comparable to the average accuracy of the instrument used (at a given, average distance to the points—about 20 m) and double measurement of the points (in two systems) gave an error of about 2.0 mm. Thus, the accuracy of the transformation in the check points is within the limits of the accuracy of the points used for transformation. The average error of the typical observation is a rather mall value and to minimize it the number of measurement series for points necessary for the transformations (centroids) should be higher. 

Thanks to the method the accuracy of the designation of the scale change coefficient gets also higher as the coefficient can be calculated or controlled many times. The proposed method can be found useful for geodetic surveys on grounds that are not stable, for example on ships or platforms where there is no possibility to (accurately) level the instrument. It can be applied also for sensors mounted on Unmanned Aerial Vehicles. The method permits an estimation of errors of the exterior orientation of sensors and thus can be applied for precise IMU calibration. The method can be also useful for big rotation angles and is a multi-realization of the Total Free Station Transformation in Hilbert space. It does not require levelling or centering of the instrument but it still allows one to maintain a high accuracy of the measurements. The MCT (MCIT) transformation is very fast, accurate and can be applied for determination of the exterior orientation of sensors.

## Figures and Tables

**Table 1:** Generating the pairs of points (vectors) in the Hilbert space (Figure 4). New sets (pairs) of points in relation to the centroids are marked in bold.

New Sets of Points	Centroid 1	Centroid 2	Centroid 3	Centroid 4	Remarks
P1 and P1′		−			$P_{1}=-P_{2}$ ${P^{\prime }}_{1}=-{P^{\prime }}_{2}$
**P2 and P2′**	+				
**P3 and P3′**	+				
**P4 and P4′**	+				
**P5 and P5′**		+			
**P6 and P6′**		+			
P7 and P7′			−		$P_{7}=-P_{3}$ ${P^{\prime }}_{7}=-{P^{\prime }}_{3}$
P8 and P8′			−		$P_{8}=-P_{5}$ ${P^{\prime }}_{8}=-{P^{\prime }}_{5}$
**P9 and P9′**			+		
P10 and P10′				−	$P_{10}=-P_{4}$ ${P^{\prime }}_{10}=-{P^{\prime }}_{4}$
P11 and P11′				−	$P_{11}=-P_{6}$ ${P^{\prime }}_{11}=-{P^{\prime }}_{6}$
P12 and P12′				−	$P_{12}=-P_{9}$ ${P^{\prime }}_{12}=-{P^{\prime }}_{9}$

**Table 2:** Laboratory data set of points.

Point No.	X [m]	Y [m]	Z [m]
1.	9.425	0.000	0.000
2.	6.686	13.244	0.000
3.	−36.856	28.020	−0.001
4.	11.137	−8.690	0.440
5.	10.632	−5.895	4.297
6.	7.908	7.072	3.788
7.	1.361	38.746	2.288
8.	−13.433	45.308	1.236
9.	−4.929	−2.337	−1.120

**Table 3:** Scaled, translated and rotated data set of points form Table 2.

Point No.	X [m]	Y [m]	Z [m]
1.	−38,184.776	134,187.409	66,568.887
2.	−46,921.266	147,381.998	72,779.882
3.	−104,603.580	148,536.384	69,313.690
4.	−32,587.744	125,247.640	62,961.972
5.	−34,647.655	125,833.266	68,589.197
6.	−43,211.548	139,031.138	74,092.271
7.	−63,983.641	171,452.844	87,275.830
8.	−84,138.956	174,094.044	85,731.157
9.	−54,122.544	127,532.728	60,415.565

**Table 4:** Points from primary system—Table 2—translated to the centroid—point no. 2.

Point No.	X [m]	Y [m]	Z [m]
1.	2.739	−13.244	0.000
2.	2.739	0.000	0.000
3.	−43.542	14.776	−0.001
4.	4.451	−21.934	0.440
5.	3.946	−19.139	4.297
6.	1.222	−6.172	3.788
7.	−5.325	25.502	2.288
8.	−20.119	32.064	1.236
9.	−11.615	−15.581	−1.120

**Table 5:** Points from secondary system—Table 3—translated to the centroid—point no. 2.

Point No.	X [m]	Y [m]	Z [m]
1.	8736.491	−13,194.589	−6210.994
2.	0.000	0.000	0.000
3.	−57,682.314	1154.386	−3466.192
4.	14,333.523	−22,134.360	−9817.909
5.	12,273.611	−21,548.732	−4190.685
6.	3709.718	−8350.859	1312.389
7.	−17,062.375	24,070.846	14,495.949
8.	−37,217.690	26,712.046	12,951.275
9.	−7201.278	−19,849.270	−12,364.317

**Table 6:** Accuracy of ICP (Independent Control Points) of MCT (MCIT) transformation in laboratory experiment.

ICP	∆X [m]	∆Y [m]	∆Z [m]
1.	$-\left(6.217\times 10^{-7}\right)$	$1.646\times 10^{-7}$	$-\left(4.396\times 10^{-7}\right)$
2.	$-\left(1.904\times 10^{-10}\right)$	$6.558\times 10^{-11}$	$-\left(2.696\times 10^{-10}\right)$
3.	$1.603\times 10^{-6}$	$6.404\times 10^{-7}$	$1.697\times 10^{-6}$
4.	$-\left(1.045\times 10^{-6}\right)$	$2.814\times 10^{-7}$	$-\left(7.081\times 10^{-7}\right)$
5.	$-\left(1.066\times 10^{-6}\right)$	$3.08\times 10^{-7}$	$-(4.676\times 10^{-7}$)
6.	$-(4.367\times 10^{-7}$)	$1.395\times 10^{-7}$	$-(5.603\times 10^{-7}$)
7.	$1.108\times 10^{-6}$	$-(2.785\times 10^{-7}$)	$9.361\times 10^{-7}$
8.	$1.759\times 10^{-6}$	$-(1.037\times 10^{-7}$)	$1.514\times 10^{-6}$
9.	$-(3.541\times 10^{-7}$)	$4.773\times 10^{-6}$	$-(1.182\times 10^{-7}$)

**Table 7:** Mean error (standard deviation) for example laboratory data sets.

No. of Data Set	ω [°]	φ [°]	κ [°]	$m_{X}$ [m]	$m_{Y}$ [m]	$m_{Z}$ [m]	$m_{P}$ [m]
1.	27.35478	5.578938	19.30716	$3\times 10^{-7}$	$1\times 10^{-7}$	$3\times 10^{-7}$	$5\times 10^{-7}$
2.	57.35478	43.578938	79.30716	$2\times 10^{-7}$	$3\times 10^{-7}$	$3\times 10^{-7}$	$4\times 10^{-7}$
3.	94.35478	199.578938	89.30716	$2\times 10^{-7}$	$4\times 10^{-7}$	$4\times 10^{-7}$	$6\times 10^{-7}$

**Table 8:** Accuracy of exterior (angular) orientation of sensor in laboratory tests (on simulated data)—maximum deviation (errors) according to relation (23).

**Value**	$m_{\omega }\;\left[{}^{\circ}\right]$	$m_{\varphi }\;\left[{}^{\circ}\right]$	$m_{\kappa }\;\left[{}^{\circ}\right]$
	$3\times 10^{-6}$	$2\times 10^{-6}$	$2\times 10^{-6}$

**Table 9:** Field surveying data with the levelled and centered instrument (Total Station).

Point No.	X [m]	Y [m]	Z [m]
1.	0.0000	0.0000	0.0000
2.	25.7233	0.0020	1.6480
3.	15.7988	6.6230	6.7835
4.	0.8898	15.7203	7.1685
5.	−16.2395	25.6953	2.2100
6.	−33.6763	−2.3131	1.4220
7.	−8.2938	−43.5918	−0.0560
8.	27.8830	−39.3765	0.0150
9.	11.7693	−2.1383	−0.0230

**Table 10:** Field surveying data with non levelled and non-centered instrument (Total Station), oblique and translated.

Point No.	X [m]	Y [m]	Z [m]
1.	3.0340	−3.3873	1.4905
2.	28.4880	0.0010	0.7743
3.	17.6575	5.2073	5.7060
4.	1.6698	12.2505	5.7813
5.	−16.5115	19.9135	0.4630
6.	−27.3055	−30.7783	−1.0235
7.	0.5850	−47.6888	−2.0020
8.	35.8825	−38.7258	−1.0260
9.	14.9838	−3.9505	−1.2465

**Table 11:** Mean error (standard deviation) for one of field surveying data set.

**Mean Errors on ICP**	$m_{X}$ **[m]**	$m_{Y}$ **[m]**	$m_{Z}$ **[m]**	$m_{P}$ **[m]**
	$2.1\times 10^{-3}$	$0.6\times 10^{-3}$	$1.3\times 10^{-3}$	$2.6\times 10^{-3}$

**Table 12:** Exterior (angular) orientation of sensor in field surveying and maximum errors.

**Value**	ω [°]	φ [°]	κ [°]	$m_{\omega }\;\left[{}^{\circ}\right]$	$m_{\varphi }\;\left[{}^{\circ}\right]$	$m_{\kappa }\;\left[{}^{\circ}\right]$
	−0.304545	−1.423586	−7.590548	$3\times 10^{-6}$	$2.4\times 10^{-6}$	$0.5\times 10^{-6}$

**Table 13:** Mean errors of transformations (standard deviation) for laboratory data set without distortion by random errors.

Transformation	Mean Errors on GCP [mm]				Mean Errors on ICP [mm]			
	$m_{X}$	$m_{Y}$	$m_{Z}$	$m_{P}$	$m_{X}$	$m_{Y}$	$m_{Z}$	$m_{P}$
MCT (MICT)	0.00	0.00	0.00	0.00	0.00	0.00	0.00	0.00
Moldensky-Badekas	0.44	0.80	0.30	0.96	0.44	0.83	0.24	0.97
Bursa-Wolf	0.80	0.00	0.00	0.80	0.80	0.00	0.13	0.81
Leica (PCMS)	0.02	0.00	0.00	0.02	0.03	0.04	0.10	0.11
TFS	0.00	0.00	0.00	0.00	0.05	0.04	0.05	0.08

**Table 14:** Mean errors of transformations (standard deviation) for laboratory data distorted by random errors according to normal Gaussian distribution.

Transformation with Standard Deviation: $\sigma_{x}=\sigma_{y}=\sigma_{z}=1.5\;\left[mm\right]$	Mean Errors on GCP [mm]				Mean Errors on ICP [mm]			
	$m_{X}$	$m_{Y}$	$m_{Z}$	$m_{P}$	$m_{X}$	$m_{Y}$	$m_{Z}$	$m_{P}$
MCT	0.00	0.00	0.00	0.00	1.89	0.92	2.15	3.01

**Table 15:** Mean errors of transformations (standard deviation) for one of field surveying data set.

Transformation	Mean Errors on GCP [mm]				Mean Errors on ICP [mm]			
	$m_{X}$	$m_{Y}$	$m_{Z}$	$m_{P}$	$m_{X}$	$m_{Y}$	$m_{Z}$	$m_{P}$
MCT (MICT)	0.00	0.00	0.00	0.00	1.83	1.27	3.55	4.19
Moldensky-Badekas	0.24	0.80	0.22	0.87	3.24	1.95	2.33	4.44
Bursa-Wolf	0.29	0.82	0.21	0.89	3.28	1.93	2.31	4.46
Leica (PCMS)	0.29	0.79	0.22	0.87	3.27	1.95	2.33	4.46
TFS	0.00	0.00	0.00	0.00	4.09	3.03	12.00	13.04

## References

[1] Kielich S. (1977). Molekularna Optyka Nieliniowa.

[2] A Note of the Bursa-Wolf and Moldensky-Badekas Transformations. https://www.researchgate.net/publication/228757515_a_note_on_the_bursa-wolf_and_molodensky-badekas_transformationsURL.

[3] Stark M. (1974). Geometria Analityczna z Wstępem do Geometrii Wielowymiarowej.

[4] Schofield W. (1984). Engineering Surveying.

[5] Soler T.A. (1998). Compendium of transformation formulas useful in GPS work. J. Geod..

[6] Susskind L., Hrabovsky G. (2014). Classical Mechanics Theoretical Minimum.

[7] Hofmann-Wellenhof B., Lichtenegger H., Collins J. (1997). Global Positioning System. Theory and Practice.

[8] Stępień G., Sanecki J., Klewski A., Zalas E. Method of parameter reduction in the transformation of oblique photographs and proposal of its implementation in Unmanned Aerial Systems. Proceedings of the IEEE Baltic Geodetic Congress (Geomatics).

[9] Baranowski L. (2013). Equations of motion of a spin-stabilized projectile for flight stability testing. J. Theor. Appl. Mech..

[10] Kurczyński Z. (2014). Fotogrametria.

[11] Zalas E., Sanecki J., Klewski A., Stępień G. (2016). Determining the spatial orientation of the remote sensing sensors on the basis of incomplete coordinate systems. Sci. J. Marit. Univ. Szczec..

[12] Jue L. (2008). Research on close-range photogrammetry with big rotation angle. Int. Arch. Photogramm. Remote Sens. Spat. Inf. Sci..

[13] Bursa M. (1962). The theory for the determination of the non-parallelism of the minor axis of the reference ellipsoid and the inertial polar axis of the Earth, and the planes of the initial astronomic and geodetic meridians from the observation of artificial Earth satellites. Studia Geophysica et Geodetica.

[14] Wolf H. (1963). Geometric connection and re-orientation of three-dimensional triangulation nets. Bull. Géod..

[15] Stępień G., Zalas E., Ziębka T. (2017). New approach to isometric transformations in oblique local coordinate systems of reference. Geod. Cartogr. J. Comm. Geod. Pol. Acad. Sci..

[16] Kojdecki M.A. (2014). Wybrane Zagadnienia Analizy Funkcjonalnej—Skrypt.

[17] Olszewski J. (1973). Nowoczesne Metody Matematyczne Fizyki.

[18] Leja F. (1969). Rachunek Różniczkowy i Całkowy.

